# Neurogenesis-Promoting Natural Product α-Asarone Modulates Morphological Dynamics of Activated Microglia

**DOI:** 10.3389/fncel.2016.00280

**Published:** 2016-12-09

**Authors:** Qing Cai, Yuanyuan Li, Jianxin Mao, Gang Pei

**Affiliations:** ^1^State Key Laboratory of Cell Biology, Institute of Biochemistry and Cell Biology, Shanghai Institutes for Biological Sciences, Chinese Academy of SciencesShanghai, China; ^2^Graduate School, University of Chinese Academy of Sciences, Chinese Academy of SciencesShanghai, China; ^3^School of Life Science and Technology, and the Collaborative Innovation Center for Brain Science, Tongji UniversityShanghai, China

**Keywords:** α-asarone, microglia, morphological dynamics, MCP-1, zebrafish, neuroinflammation

## Abstract

α-Asarone is an active constituent of *Acori Tatarinowii*, one of the widely used traditional Chinese Medicine to treat cognitive defect, and recently is shown to promote neurogenesis. Here, we demonstrated that low level (3 μM) of α-asarone attenuated LPS-induced BV2 cell bipolar elongated morphological change, with no significant effect on the LPS-induced pro-inflammatory cytokine expressions. In addition, time-lapse analysis also revealed that α-asarone modulated LPS-induced BV2 morphological dynamics. Consistently a significant reduction in the LPS-induced Monocyte Chemoattractant Protein (MCP-1) mRNA and protein levels was also detected along with the morphological change. Mechanistic study showed that the attenuation effect to the LPS-resulted morphological modulation was also detected in the presence of MCP-1 antibodies or a CCR_2_ antagonist. This result has also been confirmed in primary cultured microglia. The *in vivo* investigation provided further evidence that α-asarone reduced the proportion of activated microglia, and reduced microglial tip number and maintained the velocity. Our study thus reveals α-asarone effectively modulates microglial morphological dynamics, and implies this effect of α-asarone may functionally relate to its influence on neurogenesis.

## Introduction

Since ancient time, the applications of naturally existing herbs effectively treating memory deteriorations and cognitive declines had been well-documented in the Traditional Chinese Medicine (Park et al., 2002; Yao et al., 2009; Liu et al., 2011; Zhu et al., 2015). Modern researches on natural herbs, nowadays, endeavor to scrutinize further into cellular and molecular mechanisms. One famous example is the formulated prescription Smart Soup (SS) (Hou et al., 2014), which efficiently ameliorates memory deficit. Treatment with SS improved Alzheimer's disease (AD)-related symptoms in APP/PS1 mice. *Acori Tatarinowii* (AT) as one of the three herbs constitute the formula in particular exhibits neuron-protective effect (Lee et al., 2003; Lin et al., 2012). Further investigation in this natural herb revealed its major active constituent asarone exists as stereoisomers (α- and β-asarone), both of which promoted neurogenesis in AD model mice (Mao et al., 2015). Moreover, it was also reported that α-asarone displayed anti-inflammatory effect in the LPS-activated microglia as well as in the Parkinson's model mice (Kim et al., 2015). Such dual-functional product may serve great advantages in the treatment of AD. As it is well known that activated microglia mediates inflammation in the central nervous system (CNS) (Olson and Miller, 2004; Prinz et al., 2006; Graeber et al., 2011), and the activation of which is accompanied by changes in cell morphology and motility (Hanisch and Kettenmann, 2007; Liaury et al., 2012). Therefore, it is of our interest to further explore the effect of α-asarone on microglial behavior, including morphology and motility, both of which contribute to the anti-inflammation mechanism.

At the present, microglia morphology and motility change in response to inflammatory stimuli had been reported in many *in vitro* studies (Rangroo Thrane et al., 2012; Gyoneva et al., 2014). In addition to these findings, it is of great importance to explore how microglia behave in their endogenous niche. To achieve this, we took the advantage of zebrafish larva. The transparent larval brain and previously established transgenic lines enabled extensive visualization of fluorescently labeled microglia in living zebrafish brain. In combination with our well-established three-dimensional morphology analysis and time-lapse confocal imaging technology, *in vivo* researches of microglial dynamics in zebrafish optic tectum are achievable (Li et al., 2016).

In this study, we compared the LPS- and *E*. *coli*-induced *in vitro* and *in vivo* responses as results of α-asarone modulation. We showed α-asarone exhibited an opposing effect to the LPS-induced behavioral change, and the morphological changes may be linked to MCP-1 expression. Our results revealed that, in comparison to the pro-inflammatory cytokine expressions, microglia behavioral responses are more sensitive to changes in the inflammatory stimuli.

## Materials and methods

### Animals

The present study was performed in strict accordance with the guidelines of the Institute of Biochemistry and Cell Biology, Chinese Academy of Sciences. All experimental protocols were approved and overseen by the Animal Care and Use Committee of the Shanghai Institute of Biochemistry and Cell Biology, Chinese Academy of Sciences. All mice were maintained in pathogen-free conditions.

### Cell culture and treatment

BV2 cells were cultured and maintained in Dulbecco's Minimal Essential Medium (DMEM), supplemented with 10% fetal bovine serum (FBS) and 100 U/ml penicillin and 0.1 mg/ml streptomycin. Primary microglia cells were prepared from wild type SD rat on postnatal day 1. In brief, the mixed rat brain region including cortex and hippocampus was dissected and the meninges were carefully removed. The brain tissues were dissociated into single cells by gentle scissoring and pipetting. The resultant cell suspension was seeded to a T75 flask with DMEM supplemented with 10% FBS and 100 U/ml penicillin and 0.1 mg/ml streptomycin. Microglial cells were isolated from the astrocyte monolayer sheet by shaking.

In all cell-based assays, BV2 and primary microglia cells were pre-treated with α-asarone (Fluka) at various concentrations (0.3–100 μM) for 2 h, followed by 0.1 or 1 μg/ml LPS (055:B5, Sigma) stimulation for further 24 h.

### Immunocytochemistry

BV2 cells and cultured rat microglia cells were fixed with 4% PFA in PBS for 15 min at room temperature, followed by 30 min blocking in PBS containing 1% BSA and 0.1% Triton X-100. The cultured rat microglia cells were incubated with anti-Iba1 (1:1000, WAKO) primary antibody for 2 h at room temperature, and Cy3-conjugated anti-rabbit IgG (1:1000) secondary antibody for 1 h avoiding light exposure. BV2 cells were incubated with PE-conjugated CD86 (1:100) or APC-conjugated CD80 (1:100) antibodies overnight at 4°C, avoiding light exposure. Afterwards, cells were stained with DAPI (1:1500) for 10 min at room temperature.

### Cell viability assay

The cytotoxicity potential of α-asarone were examined in the presence and absence of LPS. BV2 cell viability was tested using the CellTiter-Glo luminescent cell viability assay (Promega) following the manufacturer's instructions.

### Scratch assay

BV2 and primary microglial cells were grown in 48-well plates till 80% confluent. The monolayer of cells was then wounded with a sterile 200 μl pipette tip, and washed three times with sterile PBS. Afterwards, the cells were incubated with α-asarone (0.3, 3, and 100 μM) in the presence and absence of LPS for 24 h. Additionally, primary microglia cells were stained with CellTrace™ CFSE Cell Proliferation Kit (Invitrogen C34554) before microscopic imaging.

### Cell imaging and morphological characterization

BV2 cells were seeded into 24-well plates at appropriate densities. The LPS and α-asarone treatments were applied according to Section Cell Culture and Treatment. The MCP-1 (Peprotech), CCR2 antagonist RS504393 (Sigma) and mouse biotinylated MCP-1 antibody (ELISA, DAKEWE Biotech Company) were applied with LPS where necessary. At the end of 24-h treatment, cell images were captured by Zeiss Observer Z1 microscope with 20X objective (Plan-Neofluar 20X/0.4). The BV2 immunocytochemistry staining images were documented using Olympus FV1200MFE microscope with UPlanSApo 40X2 (40X/0.95) objective. Primary microglia immunocytochemistry staining images were captured using Olympus IX51 camera with 20X/0.45 Olympus objective. In the Scratch assay, BV2 cells and primary rat microglia cells migrated into the open area was imaged by Zeiss Observer Z1 microscope with Zeiss Plan-Neofluar 5X and 20X objectives (Plan-Neofluar 5X/0.16, Plan-Neofluar 20X/0.4).

For morphological analysis, only the single distinct cells were selected. Dividing cells and cells attached to others were not included. Image data were processed by Fiji ImageJ software (Version 2.0.0). The following parameters were quantified: cell area, perimeter, Feret's diameter (the longest distance between any two points along the selection boundary) and circularity ($4\pi \frac{Area}{Perimeter^{2}}$). For BV2 cell CD86 and CD80 expressions, the integrated intensities of each single distinct cell were measured, the MFI (mean fluorescent intensity) levels were calculated with respect to the “Short” and “Long” morphologies.

### ELISA determination of IL-6 and MCP-1

For ELISA assays, the supernatants from each treatment were collected and stored at −20°C if not used immediately. Prior to the assay, the supernatant was centrifuged to remove cellular debris, and diluted by an appropriate factor. IL-6 and MCP-1 expression were determined using mouse-specific pre-coated ELISA kits (DAKEWE Biotech Company) according to manufacturer's instructions.

### RNA isolation and reverse transcription

Total RNA was extracted from BV2 cells using the TRI Reagent® (Sigma) following the manufacturer' s instructions. Purity and integrity of the RNA was assessed by NanoDrop 1000 Spectrophotometer (Thermo Scientific) and stored at −80°C if not for immediate use. The first-strand cDNS synthesis was performed using the TIANScript M-MLV kit (TIANGEN) following manufacturer's instruction. The synthesis was carried out with the addition of rRNasin® (Recombinant rRNasin® Ribonuclease Inhibitor, Promega).

### Quantitative real-time reverse transcription-polymerase chain reaction (qRT-PCR)

The mRNAs expression was determined by quantitative real-time PCR using the 2x HotStart SYBR Green qPCR Mater Mix kit (ExCell). Each reaction was performed in duplicates, and the supermix contained 4 μl pre-diluted cDNA and 0.25 μM primers in a total volume of 25 μl. The reaction parameters were as follow: 10-min of a 95°C cycle following 40 cycles of denaturation (95°C for 30 s), annealing (60°C for 30 s) and extension (72°C for 30 s). An additional cycle was performed for evaluation of primer's dissociation curve at 95°C for 1 min, 60°C for 30 s and 95°C for 30 s. Primer sequences used in the experiments are listed in the Supplementary Table 1.

### *In vitro* time-lapse imaging analysis of microglia morphological dynamics

BV2 cells were seeded into the 96-well glass bottom black plate. After 18 h, cells were treated with LPS and α-asarone according to Section Cell Culture and Treatment. The plate was imaged using the Cellomics ArrayScan VTI 700 (Thermo Scientific, Pittsburgh, PA, USA) with 20X objective under bright field. For each well, real-time images were captured from 16 adjacent fields at each time point, at 10-minute intervals for 24 h. During the course, cells were maintained at 37°C with 5% CO_2_. For data processing, each single distinct cell was selected. The long cell number, motility, cell area and circularity parameters were analyzed using Fiji ImageJ (version 2.0.0).

### *In vivo* time-lapse imaging analysis of motility and morphological dynamics

#### Zebrafish embryos

Zebrafish and embryo were maintained at 28.5°C in a 14/10 light/dark cycle (Kimmel et al., 1995). To inhibit pigmentation, 0.003% PTU was added at 24 h post fertilization (hpf).

#### Bacterial infections

*Escherichia Coli* (*E. coli*) strain containing pQE801 plasmid expression tdTomato were grown in LB medium with 100 μg/ml Ampicillin at 37°C. For infections, 1 ml overnight cultures were centrifuged for 3 min at 5000 rpm, and washed and re-suspended in 1 ml PBS. Bacteria were injected directly into the optic tectum of embedded larva, and images were captured 60 min after the injection, for the duration of 1 h.

#### *In vivo* time-lapse confocal imaging

For live imaging, 5-dpf larvae were embedded in 1.5% low melting-point agarose (Sigma) at 28.5°C automatic thermostats. Larvae were placed in a dorsal view without anesthetics. Images were carried out under a Zeiss 40 × NA 0.80 water immersion objective with an Olympus FV1000 upright confocal microscope (473 nm, 543 nm; Japan). The image stacks were in the range of 40–65 μm in depth, captured at 1 μm/optical section at a 5-min interval.

#### Image analysis

Resting state morphological dynamics were analyzed as previously described (Li et al., 2012, 2016). The cell area, tip number, deformation speed and migration velocity of microglia were measured by the Fiji ImageJ (version 2.0.0). The tip number is also referred to as microglial processes or brunch number. Deformation speed is defined as $\frac{\Delta Area}{\left(Area\times \Delta t\right)}$. Velocity here referred to the speed of microglia migrating toward *E*. *coli*.

### Statistics

Statistical analysis was performed using GraphPad Prism 6 software (Graphpad Software, La Jolla, CA, USA). Results were analyzed by unpaired two-tailed Student's *t*-test to determine the significance of treatment sets. For group comparisons, two-way ANOVA analysis of variance was performed. All data were presented as mean ± SEM. *P* < 0.05 was considered to have significant difference.

## Results

### α-asarone treatment attenuates LPS-induced microglia morphological changes

**Figure 1:**
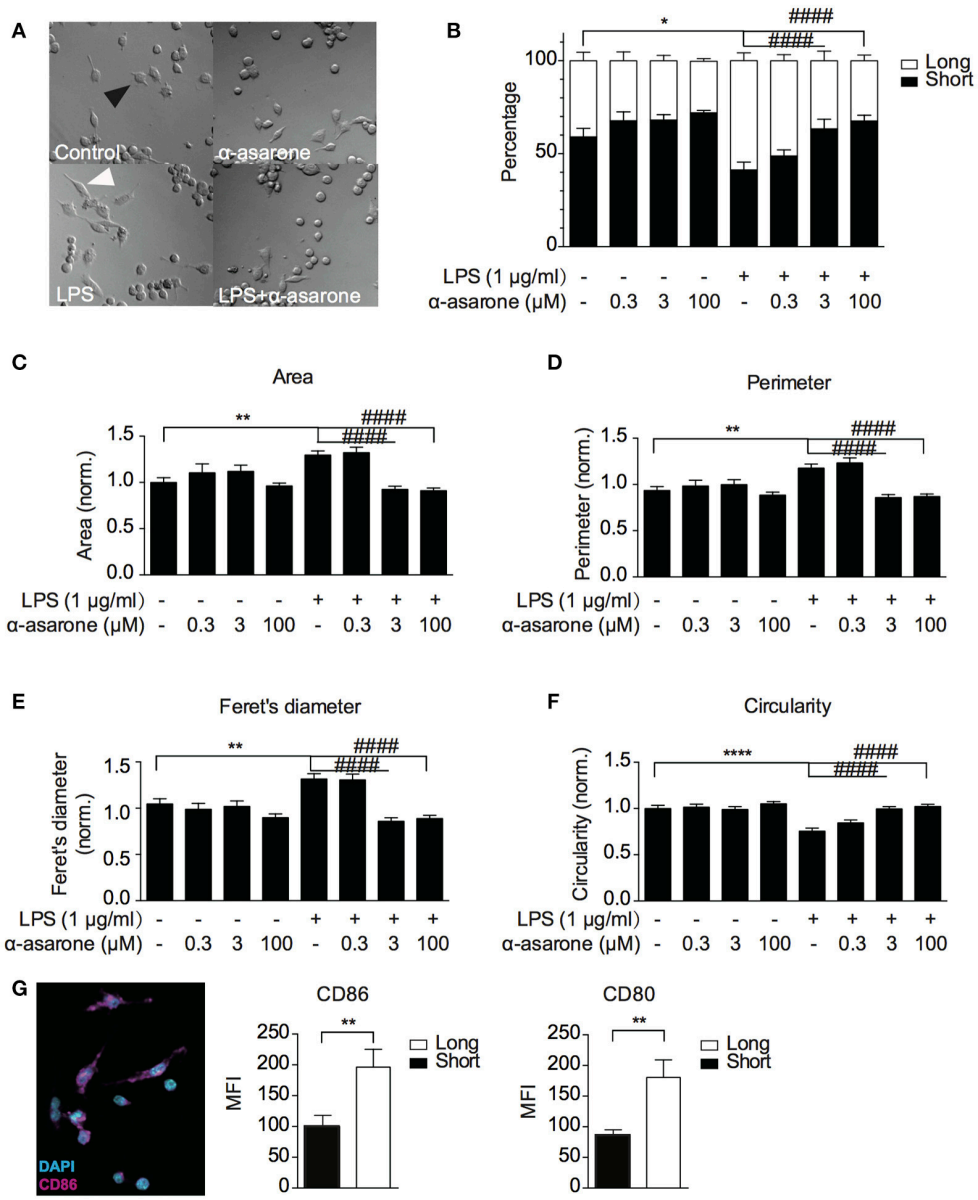
**α-asarone treatment attenuates LPS-induced microglia morphological changes**. BV2 microglia morphological changes were characterized **(A)** BV2 microglia showed differential morphologies with respect to changing treatment conditions. Black arrowhead, defined as “short” morphology, white arrowhead, as “long” morphology. **(B)** The ratio of short and long morphology in a cell population after treatments with α-asarone (0.3, 3, and 100 μM) in the presence and absence of LPS. Stimulation with LPS led to an increased population of cells in long morphology. α-asarone attenuated the LPS-induced morphological changes. **(C–F)** Four parameters characterizing microglial morphology: Area **(C)**, Perimeter **(D)**, Feret's diameter **(E)**, and Circularity **(F)**. The CD86 and CD80 activation marker expressions were determined in “long” and “short” cells **(G)**. A representative image of CD86 was shown, with DAPI in cyan and CD86 in magenta. Both CD86 and CD80 expressions were up-regulated in “long” cells. Asterisks indicates differences compared to “Control” (^*^p < 0.05, ^**^p < 0.01, ^****^p < 0.0001), crosshatches indicates differences compared to “LPS” (^####^*p* < 0.0001) two-way ANOVA for morphology comparisons between different treatment conditions, *n* = three independent experiments. For CD86 and CD80 expressions, the unpaired two-tailed Student *t*-test were used for comparison between “long” and “short” cells).

Previously, α-asarone was reported to promote neuronal progenitor cells (NPC) proliferation (Mao et al., 2015). We first confirmed that α-asarone (0.3–100 μM) did not promote BV2 microglia proliferation in the presence and absence of LPS stimulation (Supplementary Figure 1). The results also indicated that α-asarone did not exhibit cytotoxicity. Therefore, we proceeded to characterize the effect of α-asarone on microglia morphology. We examined the BV2 cell morphological changes at basal and LPS-stimulated conditions. First, we noticed the cell morphology changed with respect to differential treatment conditions (Figure 1A). We defined the “short” and “long” cells and recorded the proportion of each morphological type within the cell population. The results showed majority of cells under untreated control and α-asarone treatment alone conditions stayed short (Figure 1B). Stimulation by LPS greatly increased the number of “long” cells. However, the addition of α-asarone significantly attenuated such morphological changes in a concentration-dependent manner. We then further quantitatively defined the morphology using four parameters: the cell area, perimeter, Feret's diameter and circularity (Figures 1C–F). The results were consistent with the Figure 1B findings. Under the conditions when cells were stimulated with LPS, α-asarone treatment significantly reduced cell area, perimeter and Feret's diameter and circularity, also in a dose-dependent manner. From this, we raised the question whether these long cells correspond to the activated microglia. By immunocytochemistry assay, we examined the CD86 and CD80 expression in “long” and “short” cells after LPS and α-asarone treatments as above (Figure 1G). We found significant increases in both CD86 and CD80 expression in “long” cells compared with “short” cells, indicating that the “long” cells, indeed, correspond to the activated microglia. Therefore, these data implied that α-asarone could attenuate activated microglia morphological changes.

In addition, we analyzed the morphological changes of activated BV2 and primary microglia using a wound-healing model. As previously described, LPS induces microglia migration toward a scratched wound (Karlstetter et al., 2010, 2011; Lively and Schlichter, 2013). We created a single uniform scratch on the monolayer of cells (Supplementary Figures 2A, 3A). After migrating toward the open wound, the morphology of each distinct cell within the wounded area was analyzed. We also determined the proportion of “long” and “short” cells (Supplementary Figure 2B), and quantitatively defined the cell area, perimeter, Feret's diameter and circularity (Supplementary Figures 2C–F, 3B–E). The results were consistent with Figure 1 findings. For primary cultured microglia, no change in cell circularity was detected.

### Time-lapse analysis of LPS-induced microglia morphological dynamics affected by treatment with α-asarone

**Figure 2:**
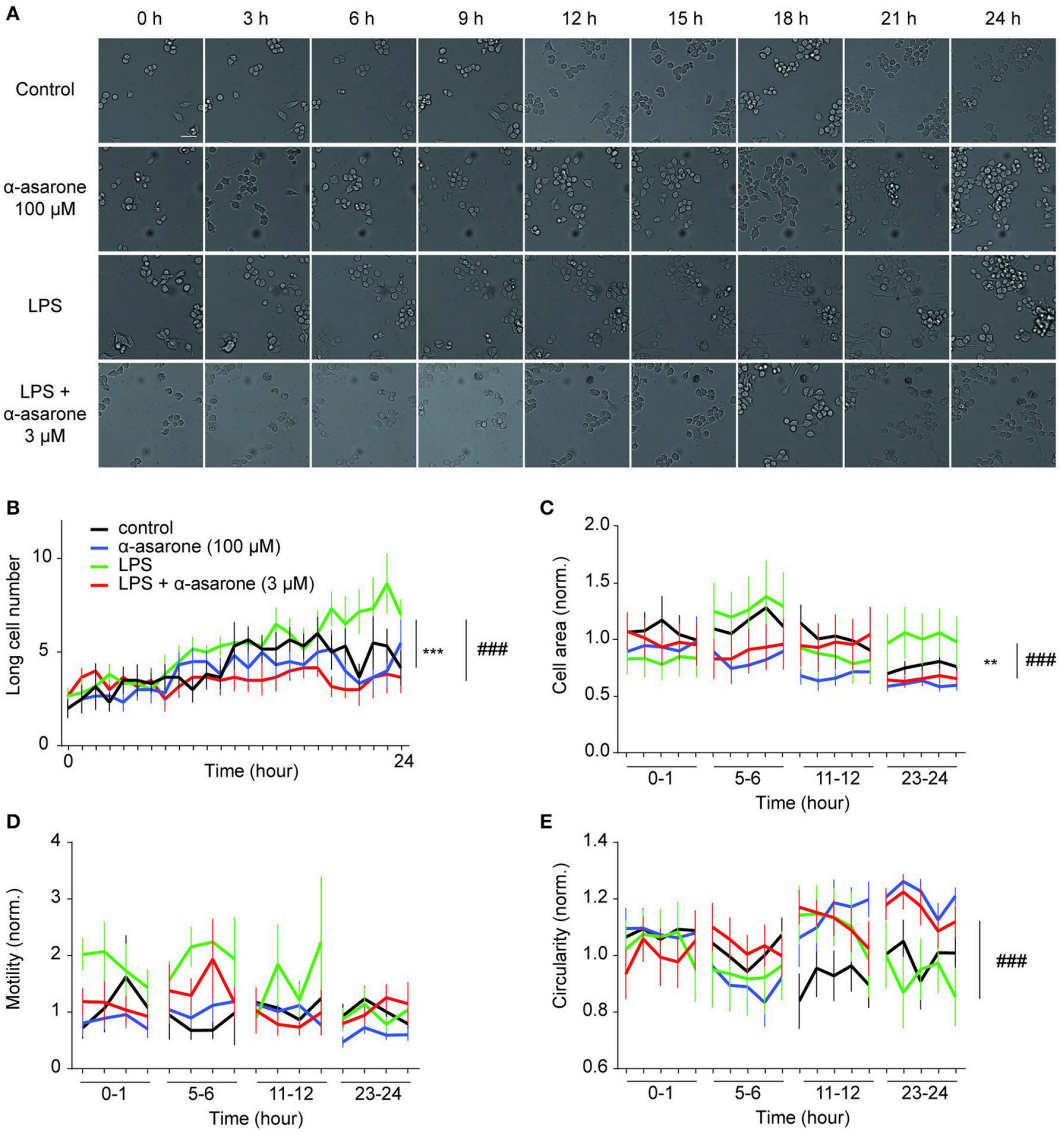
**Time-lapse analysis of LPS-induced microglial morphological dynamics affected by treatment with α-asarone**. Microglia morphological dynamics were analyzed over a 24-h time course by Cellomics ArrayScan. Images were captured at 10-min intervals. Representative images were shown for each treatment condition **(A)**. Basal level morphological modulations by 100 μM α-asarone were examined. LPS stimulation resulted in an increase in the number of long cells over time, whilst α-asarone (3 μM) stabilized the increase **(B)**. The differences were most significant at the end of 24 h. The cell area **(C)** and circularity **(E)** were determined at four representative time phases. Results were normalized to “Control.” The effect of α-asarone on the LPS-induced morphological changes was most significant at “23–24”-h time phase. In addition, LPS displayed a trend to increase cell motility compared with α-asarone (3 μM) pre-treatment condition **(D)**. Asterisks indicates differences compared to “Control” (^**^p < 0.01, ^***^p < 0.001), crosshatches indicates differences compared to “LPS” (^###^*p* < 0.001) two-way ANOVA test for at least three independent data comparisons, *n* ≥ 3).

We further examined the effect of α-asarone on BV2 microglia morphological dynamics on a 24-h time course. BV2 cells were pre-treated with 3 μM α-asarone in the presence of LPS or with 100 μM α-asarone alone. Micrographic images were documented at 10-min intervals for 24 h. For each well of a specific treatment condition, we captured images from 16 adjacent fields at each time point. Only data for typical time points were shown in Figure 2. Stimulation by LPS resulted in an increasing trend of long cell numbers. The attenuation effect of α-asarone was displayed from 7 h onwards (Figure 2B). The differences between the two treatment conditions were most significant at 24 h. This was consistent with earlier findings (Figure 1). We also determined the cell area and circularity parameters (Figures 2C,E). Changes in both parameters were monitored for 1 h and the results were normalized to the average of “control.” Only representative data at four specific time-phases were shown. At 23–24 h time-phase, we detected reduction in cell area and an increase in the circularity ratio related to the effect of α-asarone on the LPS-activated cells. In addition, at 5–6 h time phase, α-asarone showed a tendency to reduce cell area as well as to increase cell circularity in comparison to LPS stimulation alone. However, no statistical significance was detected. There was also no significant difference in motility, which may due to large variance in the data (Figure 2D). Time-lapse imaging consistently showed that α-asarone attenuated LPS-induced microglia morphological changes at 3 μM. A representative video was provided in the supplementary material (Supplementary Video), on the left panel cells were stimulated with LPS, and the right panel the LPS-activated cells were incubated in the presence of 3 μM α-asarone.

### MCP-1 correlates with α-asarone modulation of morphological dynamics

**Figure 3:**
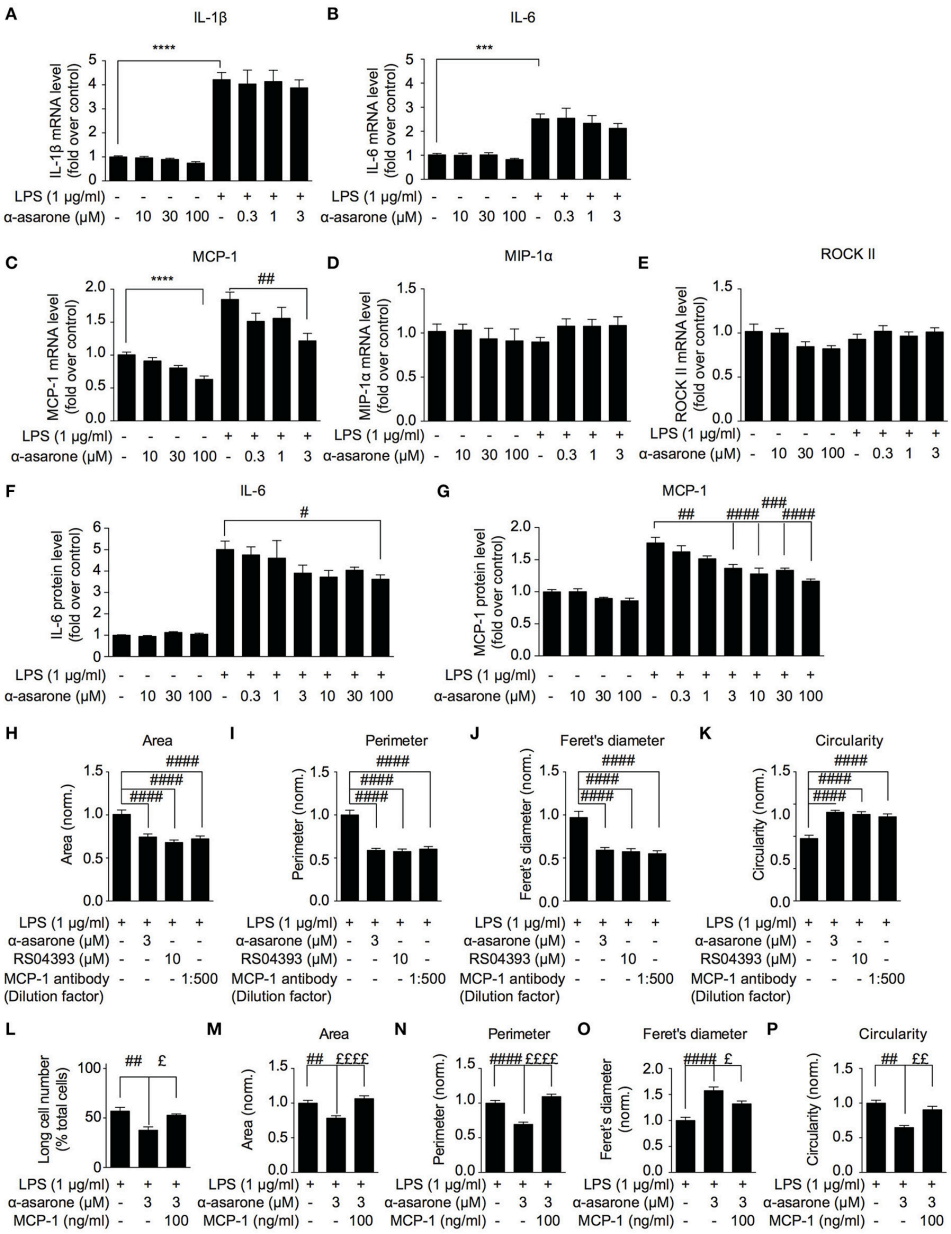
**MCP-1 correlates with α-asarone modulation of morphological dynamics**. The effect of α-asarone on the BV2 microglia pro-inflammatory cytokine **(A,B,F)** and chemokine **(C–E,G)** expressions were determined. α-Asarone (3 μM) significantly inhibited the LPS-induced MCP-1 production (qPCR, **C** and ELISA, **G**). Low concentrations of α-asarone (1–3 μM) did not inhibit the pro-inflammatory cytokine expressions **(A,B)**. Higher concentration of α-asarone (30 and 100 μM) showed inhibitory effect on the pro-inflammatory cytokine expression **(F)**. MCP-1 depletion (MCP-1 antibody) or antagonizing CCR_2_ receptors (RS504393) modulated BV2 morphology **(H–K)**, the effects were comparable to α-asarone. The MCP-1 (100 ng/ml) rescued the LPS-induced BV2 morphological changes in the presence of α-asarone **(L–P)**. Changes in the number of long cells were recorded **(L)**, and microglia morphology was characterized: cell Area **(M)**, Perimeter **(N)**, Feret's diameter **(O)**, and Circularity **(P)**. Asterisks indicates differences compared to “Control” (^***^p < 0.001, ^****^ *p* < 0.0001), crosshatches indicates differences compared to “LPS” (^#^*p* < 0.05, ^##^*p* < 0.01, ^###^*p* < 0.001, ^####^*p* < 0.0001), pound symbol indicates differences compared to “LPS + a-asarone” (^£^*p* < 0.05, ^££^*p* < 0.01, ^££££^*p* < 0.0001) two-way ANOVA test for three independent data comparisons, each performed in duplicates, *n* = 3).

Previous study reported that between 50 and 250 μM, α-asarone exhibited anti-inflammatory effect on the LPS-stimulated microglia cells (Kim et al., 2015). Nevertheless, as demonstrated in Figure 1, the minimum effective concentration of α-asarone required to modulate the morphology of activated microglia was 3 μM, which is significantly lower compared to the previous report. In order to determine whether the morphological modulations of the activated microglia were associated to the attenuation of inflammatory responses, we analyzed the expression of various pro-inflammatory cytokines. We found that 3 μM α-asarone did not inhibit the pro-inflammatory cytokine IL-1β and IL-6 mRNA expressions (Figures 3A,B). Protein level detection by ELISA showed consistent results. Increasing α-asarone concentration (above 30 μM) indeed resulted in the inhibition of LPS-stimulated IL-6 expression (Figure 3F). In addition, we confirmed these findings using 0.1 μg/ml LPS as in the previous report (Kim et al., 2015) (Supplementary Figure 4). Our results were also consistent with the previous study, such that at high concentrations (100 and 300 μM) α-asarone significantly reduced the LPS-induced IL-1β and IL-6 expressions. Meanwhile, lower concentrations of α-asarone (1 and 3 μM) did not affect IL-1β and IL-6 levels, consistent with Figures 3A,B. These results implied that the morphological modulation of activated microglia might not associate with the attenuation of inflammatory responses.

In order to define a mechanism by which α-asarone modulated microglia morphological dynamics, we analyzed the expression of MCP-1, MIP-1α and ROCKII, which were reported to influence cell migration and morphology (Xu et al., 1996; Lentzsch et al., 2003; Widera et al., 2004; Yoneda et al., 2012). The results showed that MCP-1 expression was reduced by α-asarone in a concentration-dependent manner (Figures 3C,G). The inhibition of LPS-induced MCP-1 accumulation was observed when exposed to both low (1–3 μM) (Figures 3C,G, 3 μM) and higher concentrations (10–100 μM) (3G, 10, 30, and 100 μM) of α-asarone. On the other hand, no significant changes in MIP-1α and ROCKII expressions where detected (Figures 3D,E). Therefore, MCP-1 was associated with the LPS-induced morphological changes, and α-asarone may attenuate such morphological change by regulation of MCP-1 expression. By using the CCR_2_ antagonist (10 μM RS504393) or MCP-1 antibody, we confirmed on the inhibitions of LPS-induced morphological changes (Figures 3H–K).

Inversely, the addition of MCP-1 (100 ng/ml) retained the LPS-resulted microglia morphological change in the presence of α-asarone. We recorded an increase in the long cell numbers associated with MCP-1 even in the presence of α-asarone treatment (Figure 3L). Concomitantly, changes in cell area, perimeter, Feret's diameter and circularity parameters together demonstrated an opposing effect of MCP-1 to α-asarone (Figures 3M–P). These results served clear indications that the attenuation by α-asarone to LPS-induced morphological changes was effectively rescued by MCP-1.

### Modulation by α-asarone on LPS-induced morphological changes is rescued by MCP-1 in primary microglia

**Figure 4:**
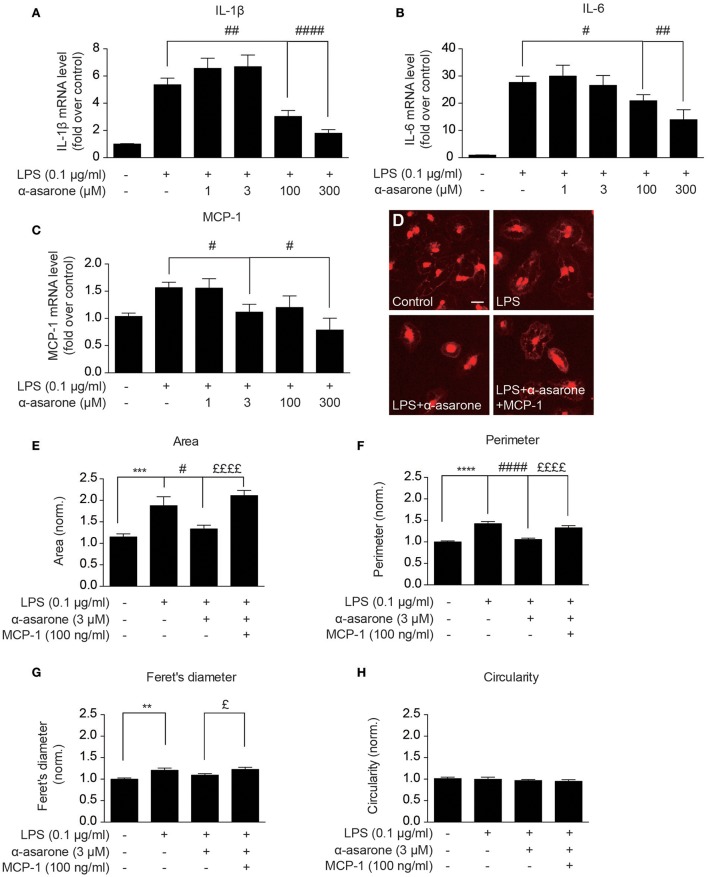
**Modulation by α-asarone on LPS-induced morphological changes is rescued by MCP-1 in primary microglia**. The effect α-asarone treatment on the 0.1 μg/ml LPS-induced IL-1β **(A)**, IL-6 **(B)**, and MCP-1 **(C)** expression in rat primary microglial cells. The microglial morphological changes were characterized after immunocytochemistry staining. Untreated microglia cells showed smaller somata **(D)**. Four parameters define microglial morphology: cell Area **(E)**, Perimeter **(F)**, Feret's Diameter **(G)**, and Circularity **(H)**. Asterisks indicates differences compared to “Control” (^**^p < 0.01, ^***^p < 0.001, ^****^p < 0.0001), crosshatches indicates differences compared to “LPS” (^#^*p* < 0.05, ^##^*p* < 0.01, ^####^*p* < 0.0001), pound symbol indicates differences compared to “LPS + a-asarone” (^£^*p* < 0.05, ^££££^*p* < 0.0001) two-way ANOVA test for five independent data comparisons, *n* = 5).

In order to validate our findings, we examined the morphological changes in primary rat microglia. We first confirmed the α-asarone effect on IL-1β, IL-6, and MCP-1 expressions (Figures 4A–C). The results showed that at higher concentrations (100 and 300 μM), α-asarone inhibited the accumulation of LPS-induced IL-1β, IL-6, and MCP-1 expressions. At lower concentration (3 μM), α-asarone specifically reduced the LPS-induced MCP-1 expression. These results correlated with the findings in BV2 cells. Subsequent morphological analysis revealed that untreated microglial cells maintained relatively small somata sizes, as defined by the cell area (Figures 4D,E). Stimulation by LPS resulted in a significant enlargement in cell area, and the reverse effect was observed when α-asarone was applied. Nevertheless, α-asarone attenuation on the LPS-induced somata enlargement was effectively rescued by MCP-1 (Figures 4D,E). We also detected significant increase in cell perimeter and Feret's diameter in the presence of LPS stimulation (Figures 4F,G). In addition, co-treatment with α-asarone showed a decreasing trend in both parameters, but the difference for Feret's diameter measurement was not significant. In addition MCP-1 rescued cell perimeter and Feret's diameter in the presence of α-asarone. Finally, no significant changes in cell circularity were detected under all conditions (Figure 4H). These results confirmed that α-asarone attenuation on LPS-induced morphological changes was rescued by MCP-1.

### α-asarone affects microglia morphological dynamics *in vivo*

**Figure 5:**
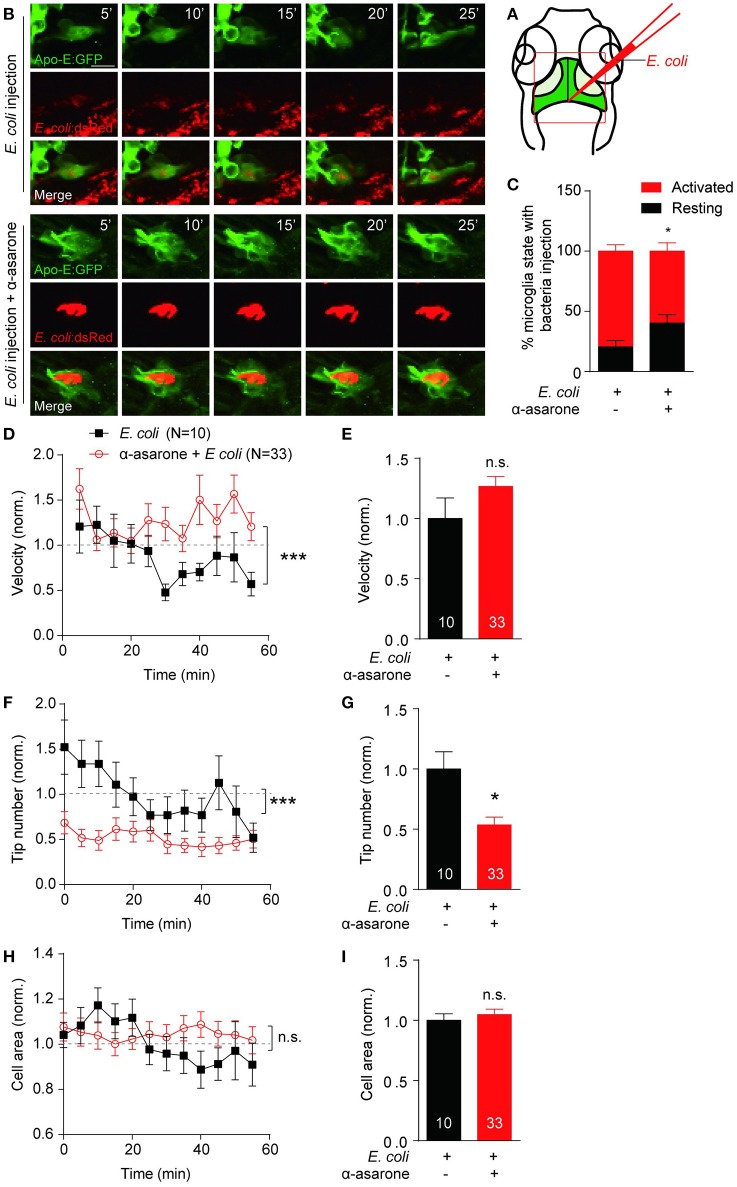
**α-asarone affects microglial morphological dynamics *in vivo***. *E*. *coli* was inject into the gap the optic tectum region **(A)** of the transgenic *Tg(Apo-E:eGFP)* zebrafish. Time-lapse images were taken at 5-min intervals **(B)**. Zebrafish pre-incubated with α-asarone showed decreased activation, *n* = 3 **(C)**. The pre-treatment did not cause significant effect to the average migration velocity **(E)**, but the dynamics of velocity change was significant **(D)**. Results were normalized to “0 min” value. α-asarone treatment resulted significant declines in both dynamical changes and average numbers of tips **(F,G)**. α-asarone treatment did not affect the cell area **(H,I)**. (^*^p < 0.05, unpaired two-tailed Student *t*-test for independent data comparison vs. *E*. *coli* injection; ^***^p < 0.001, two-way ANOVA for group comparison between the dynamical trends, the numbers were on the bars).

To determine the role of α-asarone in microglia *in vivo*, transgenic line *Tg(Apo-E:eGFP)* with microglia in green was used (Li et al., 2012). First of all, zebrafish embryos were treated with 0.3–3 μM α-asarone from 12 hpf to 5 dpf, and no developmental defects were observed under these concentrations (Supplementary Figure 5A). Therefore, we treated zebrafish embryos with 3 μM α-asarone in the presence and absence of E *coli* from 4 dpf for 24 h, and examined the morphology and dynamics of only resting microglia. The *in vivo* time-lapse imaging was carried out at 5-min intervals for 60 min according to previous study (Li et al., 2016). No significant difference was observed in the resting microglia cell area, deformation speed and tip number (Supplementary Figures 5B–G). Subsequently, we analyzed the activated microglia dynamics by injection of the red-fluorescent *E*. *coli* into the gap at the optic tectum region (Figure 5A), and images were then captured (Figure 5B). Following the previous description for microglia states (Kettenmann et al., 2011), we examined the percentage of active-state microglia in the presence of α-asarone. The results showed a remarkable decrease (control vs. α-asarone 79.4 ± 5.3 vs. 59.6 ± 6.9, *p* < 0.05, Figure 5C). Quantitative analysis of active microglia morphological dynamics showed that the effect of α-asarone to the average velocity of microglia cells was not statistically significant. However, the effect in the overall trend was significant, which indicated α-asarone maintained the activated microglial migration velocity (*p* < 0.01, Figures 5D,E). Further analysis revealed that α-asarone treatment led to approximately 50% decrease in the average tip number (asarone 53% ± 7% of control, *p* < 0.05, Figure 5G) without changes in the cell area (asarone 105% ± 4% of control, *p* = 0.55, Figures 5H,I). In addition, the dynamical changes of tip numbers change over the 60-minute time course also showed significant difference with relation to α-asarone treatment (*p* < 0.0001, Figure 5F). These results suggested that α-asarone might affect the microglia activation and function without modulating the resting state dynamics.

## Discussion

In this study, we showed the anti-inflammatory effects of a natural product and sought to unravel the underlying link between its anti-inflammation and the previously studied neurogenesis-promoting effect. Uncontrolled neuroinflammation accompanied with aberrant neurogenesis were observed in the pathogenesis of neurodegenerative diseases. Interestingly, a number of clinical drugs exhibit dual functions, and simultaneously modulate neuroinflammation and neurogenesis (Malberg et al., 2000; Kotani et al., 2006; Tchantchou et al., 2007; Hwang et al., 2010; Ji et al., 2014; Wan et al., 2016). The use of these “dual-functional” drugs might bring a greater advantage in targeting neurodegenerative diseases. In addition to these, other drugs may exhibit anti-inflammatory and anti-mitochondrial impairment functions, which sequentially serve as effective neuroprotective reagents (Xu et al., 2013; Chen et al., 2015; Wei et al., 2016). For example, simvastatin (Xu et al., 2013; Gao et al., 2016; Zhou et al., 2016) and memantine (Xu et al., 2012; Wei et al., 2016) used in Parkinson's diseases, attenuate the 6-OHDA-induced inflammation and Nur77 cytosolic translocation. The consequences of which eventually lead to suppression of neurodegeneration and to promote survival signaling. As previously described by Kim et al. (Kim et al., 2015), α-asarone suppressed the production of pro-inflammatory cytokines by LPS-activated microglia in the Parkinson's disease model mice. On the other hand, Mao et al. (Mao et al., 2015) showed that α-asarone promoted adult hippocampal NPC proliferation. Therefore, we chose α-asarone in our research conceptually as a representation of compounds which simultaneously possessing dual functions.

Besides of pro- and anti-inflammatory cytokines produced by microglia, changes in microglia behavior in terms of motility and morphological dynamics are also implicated in the pathological conditions. During inflammation, microglia change morphology frequently, and activation of microglia leads to movement toward the site of injury and inflammation (Lull and Block, 2010; Saijo and Glass, 2011). Interestingly, we found differential effects of α-asarone on pro-inflammatory cytokine production and microglia behavior when applied at distinctive doses. At low concentration (3 μm), α-asarone modulated LPS- or *E. coli*—stimulated microglia morphological dynamics as well as attenuated activation, yet no significant effect on the pro-inflammatory cytokine expressions was detected. We used both *in vitro* and *in vivo* models to confirm this finding. The changes in the activated BV2 morphologies were most obvious. In the primary cultured microglia cells, we detected limited change in the Feret's diameter and circularity. This might due to the fact that the primary microglia adapt more “irregular” and “varied” morphologies compared to BV2 cells. The morphologies of primary microglia may not simply be described as “short” and “long”. In the zebrafish model, we used *E*. *coli* to mimic the response of LPS. α-asarone attenuated activation of microglia in the *in vivo* model, which is consistent with our *in vitro* findings (Figure 1) as the number of “long” cells was also greatly reduced. Even though we did not detect changes in cell area, reduction in the microglia tip numbers indicated significant changes in cell morphology. Such modulation may provide implication for reduced phagocytic probability, which in turn may contributes to attenuation of microglia activation. When applied at high concentrations (30–100 μM), α-asarone, indeed, suppressed the pro-inflammation cytokine production, which is consistent with the previous report. These data implied that microglia morphological changes might be a more sensitive and rapid response to environmental stimulation. Therefore, microglia behavior changes may serve as an early indication for anti-inflammatory actions of therapeutic agents.

Secondly, we observed a link between the expression of MCP-1 and the effects of α-asarone on microglia morphological dynamics. We recorded a dose-dependent modulation in the LPS-induced MCP-1 level by α-asarone. Further, reduction in MCP-1 expression led to changes in the microglia morphological dynamics similar to α-asarone treatment, whilst the addition of MCP-1 rescued the LPS-induced morphological changes even in the presence of α-asarone. Therefore, α-asarone modulation of the LPS-induced microglia morphological changes may be a result of regulation of MCP-1 expression. MCP-1 was reported to enhance chemotaxis of cells toward site of inflammation and injuries (Ajuebor et al., 1998; Ashida et al., 2001; Arefieva et al., 2005; Hoh et al., 2011). Besides, MCP-1 contributes to the actin cytoskeleton rearrangement and redistribution of the tight junction protein such as occludin and claudin-5 (Stamatovic et al., 2003; Song and Pachter, 2004; Lee et al., 2009; Strecker et al., 2013), which are closely related to morphology changes. MCP-1 expression is mainly regulated by NF-κB/IκB, p38/ERK1/2 and Akt pathways, although this may also be influenced by the ambient cytokine concentrations (Takaya et al., 2003; Skalniak et al., 2009). The accumulation of MCP-1 in turn plays a positive feedback role (Werle et al., 2002; Skalniak et al., 2009; Yang et al., 2014), enabling a well-balanced and precise regulation for MCP-1. The mechanism by which α-asarone down-regulates the LPS-induced microglia pro-inflammatory cytokine release might overlap with MCP-1 regulatory pathways on NF-κB/IκB. It thus explains the α-asarone inhibition of MCP-1 accumulation.

Microglia are one of the major components of NPC niche and regulate neurogenesis in adult hippocampus (Gemma and Bachstetter, 2013; De Lucia et al., 2016). In the physiological conditions, surveillance microglia secrete trophic factors, such as the Insulin-like growth factor and Brain-derived neurotrophic factor, both of which are essential to neurogenesis (Lazarini et al., 2012; Matsuda et al., 2015). However, under pathological conditions, activation of microglia produces pro-inflammatory cytokines that lead to aberrant neurogenesis. At the concentration, which Mao et al previously reported to promote NPC proliferation (1–10 μM), we did not observe changes in the pro-inflammatory cytokines expression by α-asarone treatment. Therefore, it needs to be determined whether modulations in microglia morphology and MCP-1 expressions functionally correlate with NPC proliferation and neurogenesis, and vice versa. Interestingly, it was reported that MCP-1 exhibited neuroprotective function (Eugenin et al., 2003) and restored the number of retinal ganglion cell in the experimental glaucoma model with the concentration of MCP-1 being rather critical (Chiu et al., 2010). This probably serves an explanation to the tight regulation of MCP-1 expression. Finally, although it has not to date been generally confirmed whether most “dual-functional” drugs and compounds also influence microglia behavior, they might represent an innovative approach against neurodegenerative diseases.

## Author contributions

GP substantially controlled study conception and design, interpretation of data, and critical revision of the manuscripts for important intellectual content. QC and YL contributed equally toward this study. QC performed all *in vitro* assays and data analysis. YL performed *in vivo* zebrafish experiments, and processed and analyzed data from *in vitro* and *in vivo* time-lapse imaging. JM contributed manuscript editing and revision. QC and YL both contributed to manuscript preparation. All the authors read and approved the final article.

## Funding

This research was supported by the Ministry of Science and Technology (2015CB964502), and Shanghai Municipal Commission for Science and Technology (15JC1400202, 14DZ1900402).

### Conflict of interest statement

The authors declare that the research was conducted in the absence of any commercial or financial relationships that could be construed as a potential conflict of interest.

## Abbreviations

AD: Alzheimer's disease
APP: amyloid precursor protein
AT: *Acori Tatarinowii*
CCR2: C-C chemokine receptor type 2
CNS: central nervous system
dpf: day post fertilization
*E*. *coli*: *Escherichia coli*
ELISA: enzyme-linked immuosorbent assay
hpf: hour post fertilization
IL-1β: Interleukin -1 beta
IL-6: Interleukin -1 6
LB: Luria Bertani
LPS: lipopolysaccharides
MCP-1: monocyte chemoattractant protein
MIP-1α: macrophage inflammatory protein 1-alpha
NPC: neural progenitor cell
PS1: preseninlin 1
qPCR: quantitative polymerase chain reaction
ROCK II: Rho associated coiled-coil containing protein kinase II
SS: smart soup
Tg: transgenic.
