# Supplementary material for: Neurogenesis-Promoting Natural Product α-Asarone Modulates Morphological Dynamics of Activated Microglia
Source: Front Cell Neurosci. 2016 Dec 9;10:280. doi: 10.3389/fncel.2016.00280 (PMC5145874; doi:10.3389/fncel.2016.00280)
Supplement: Supplementary file 2 [file DataSheet1.DOCX]

Supplementary Material

Neurogenesis-promoting natural product α-asarone modulates lipopolysaccharides-induced microglial morphological dynamics

Qing Cai^a,b†^, Yuanyuan Li ^a,b,†^, Jianxin Mao ^a,b^ and Gang Pei^a,c,*^

*** Correspondence:** Gang Pei: gpei@sibs.ac.cn

# Supplementary Figures and Tables

## Supplementary Figures

## Supplementary Table


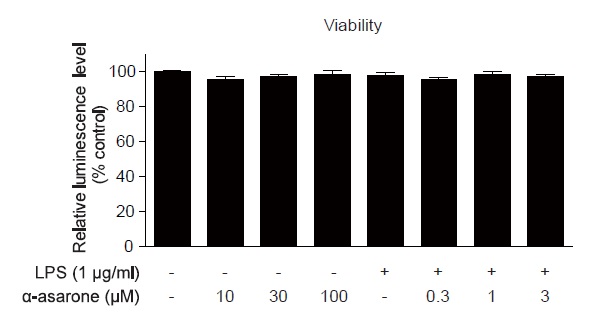


**Supplementary Figure 1.** **Examination for α-asarone proliferation and cytotoxicity potential**

BV2 microglial cell proliferation and viability levels were measured using CellTiter-Glow assay. Cells were treated with various concentrations of α-asarone in the presence or absence of LPS. Pre-incubations were carried out prior to LPS. No significant changes in the luminescence level were detected.

Supplementary Figure 2. α-Asarone attenuates LPS-induced BV2 morphological changes in the scratched wound model

BV2 microglia morphological changes were characterised after migration into an open wound. (A) Left: representative images of scratch assay, BV2 cells exposed to scratch were treated under various conditions; Right: Magnified images of scratch assay (Left), BV2 differential morphologies with respect to changing treatment conditions. Black arrowhead, “short” morphology, white arrowhead, “long” shape. (B) The ratio of short and long morphology in a cell population after the treatment with α-asarone (0.3 µM, 3 µM and 100 µM) in the presence and absence of LPS. (C-F) cell Area, Perimeter, Feret’s diameter and Circularity were determined. Changes in the ratio of short and long morphology are consistent with the differences in these parameters. (* = vs. ‘Control’; # = vs. ‘LPS’; **p* < 0.05, ***p* < 0.01, ****p* < 0.001, *****p* < 0.0001; two-way ANOVA test for three independent data comparisons, n = 3 , each performed in duplicates)

Supplementary Figure 3. α-Asarone attenuates LPS-induced primary microglia morphological changes in the scratched wound model

Primary cultured microglia morphological changes were characterised after migration into an open wound. (A) Left: representative images of scratch assay, primary microglia cells exposed to scratch were treated under various conditions; Right: microglia showed differential morphologies with respect to change conditions. Cells were stained with CFSE for optimal visualisation and imaging. α-Asarone was applied at various concentrations (0.3 µM, 3 µM and 100 µM) in the presence and absence of LPS. (B-F) Four parameters define microglial morphology: cell Area (C), Perimeter (D), Feret’s diameter (E) and Circularity (F). (* = vs. ‘Control’; # = vs. ‘LPS’; **p* < 0.05, *****p* < 0.0001; two-way ANOVA test for three independent data comparisons, n = 3 , each performed in duplicates)


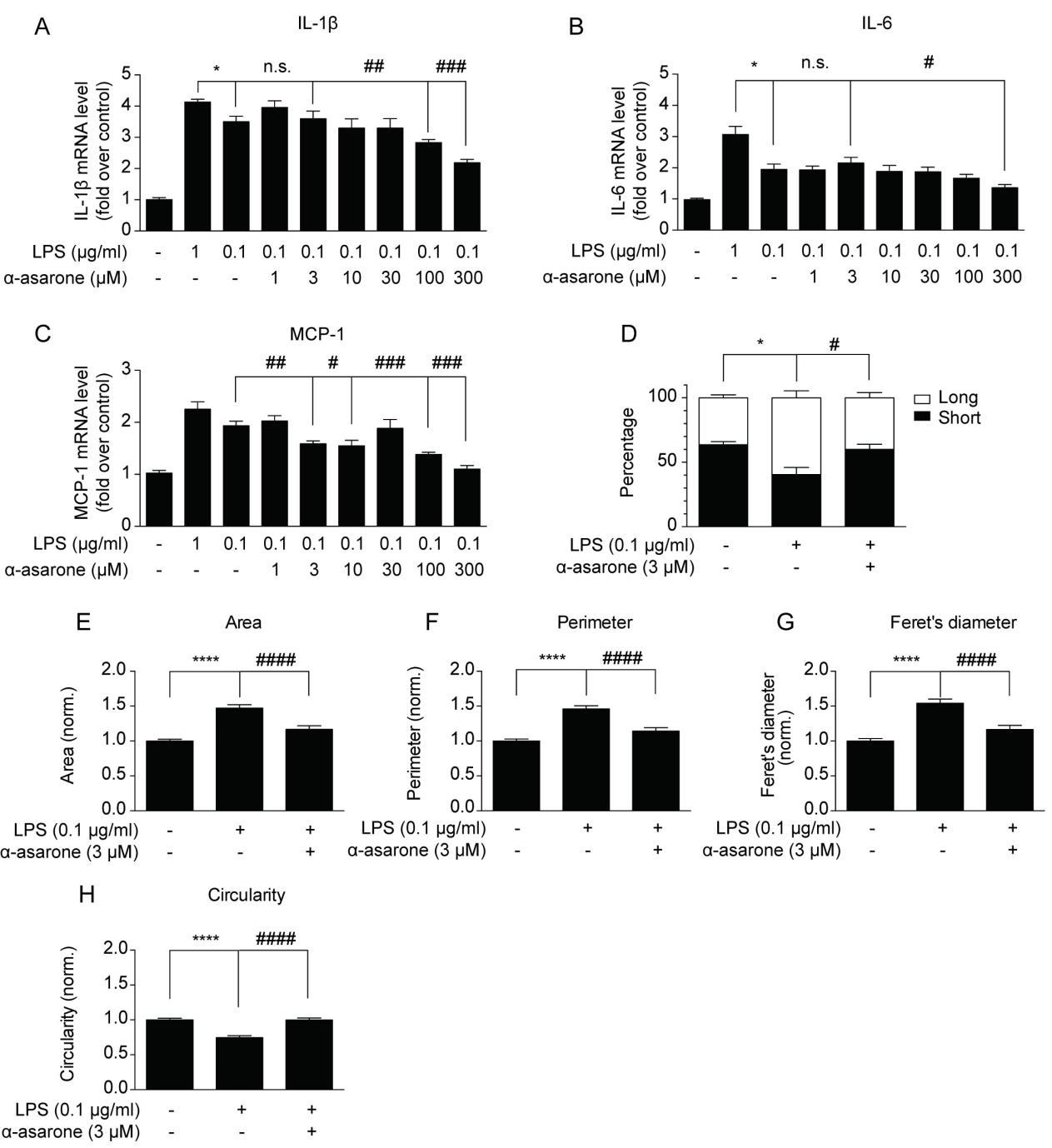


**Supplementary Figure 4.** **Examination of α-asarone effect on 100ng/ml LPS-stimulated BV2**

The effect of α-asarone on the BV2 microglia pro-inflammatory cytokine (A and B) and chemokine (C) expressions were determined. α-Asarone (3 µM) significantly inhibited 0.1µg/ml LPS-induced MCP-1 production (C). Low concentrations of α-asarone (1-3 µM) failed to inhibit pro-inflammatory cytokine expressions (A and B). BV2 microglia morphological changes were characterised. The effect of α-asarone on the ratio of short and long cells was determined in the presence of LPS (D). Cell Area (E), Perimeter (F), Feret’s Diameter (G) and Circularity (H) were determined. Changes in the ratio of short and long morphology were consistent with the differences in these parameters. (* = vs. ‘1µg/ml LPS’; # = vs. ‘100ng/ml LPS’; **p* < 0.05, ***p* < 0.05, ****p* < 0.001, *****p* < 0.0001; unpaired two-tailed Student *t*-test for three independent data comparisons, each performed in duplicates)


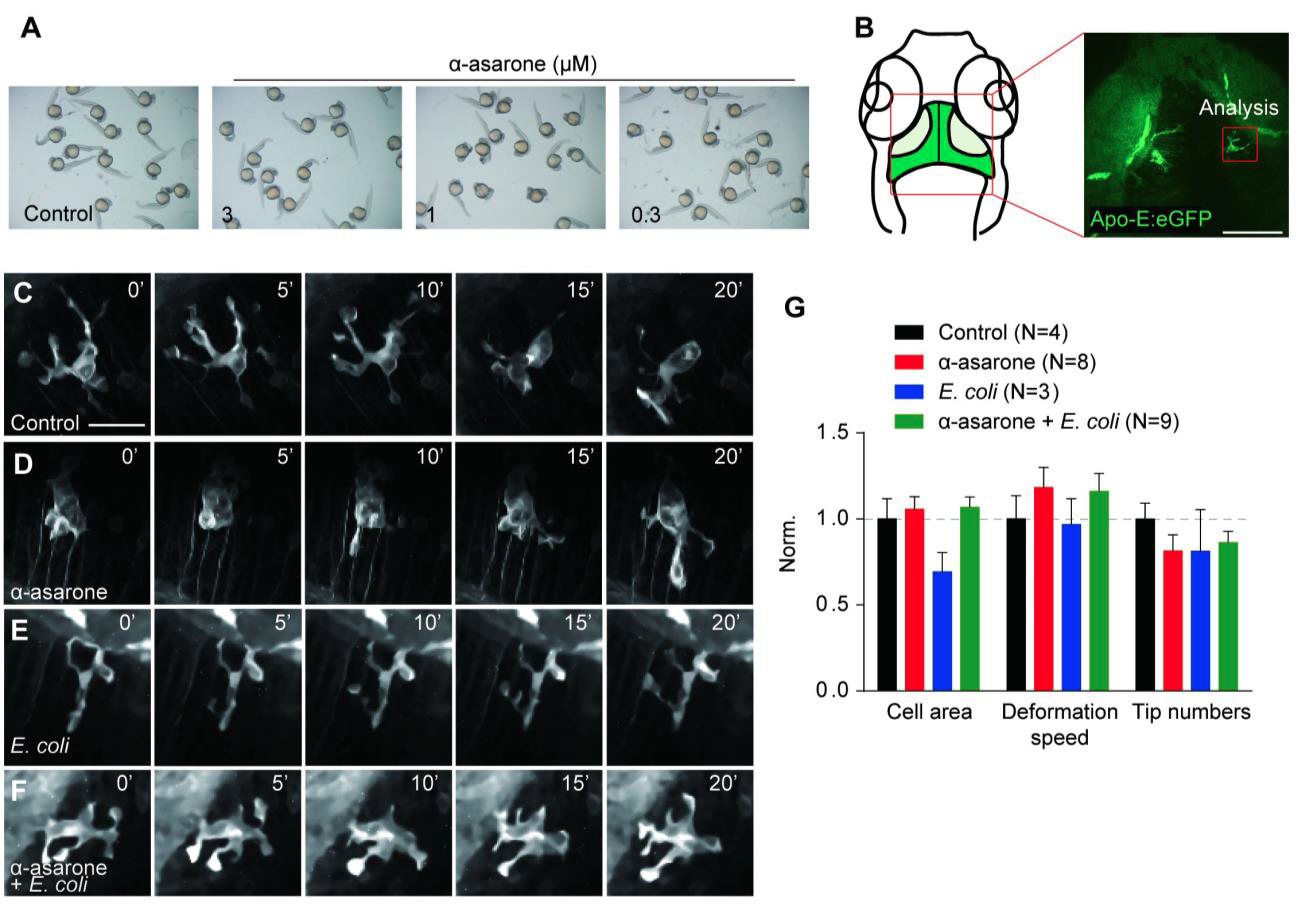


Supplementary Figure 5. Effect of α-asarone and E. *coli* on the zebrafish embryonic development and resting microglial morphological dynamics

The *Tg(Apo-E:eGFP)* transgenic zebrafish embryos were treated with 0.3-3 µM α-asarone from 12 hours post fertilisation (hpf). The developmental status was examined at 5 days post fertilisation (dpf) and revealed no defect under α-asarone treatment (0.3-3µM) (A). Real-time live imaging of microglia cells from the optic tectum region of a 5-dpf larva (B) and captured morphological dynamics of resting microglia at 5-minute intervals. (C) Untreated, (D) 3µM α-asarone, (E) E. *coli* injection, (F) E. *coli* and α-asarone (3µM) co-treatment. The results showed no significant effect of E. *coli* and α-asarone to the resting microglial cell area, deformation speed and tip numbers (G).

Supplementary Table 1. Primer pairs for qPCR analysis.


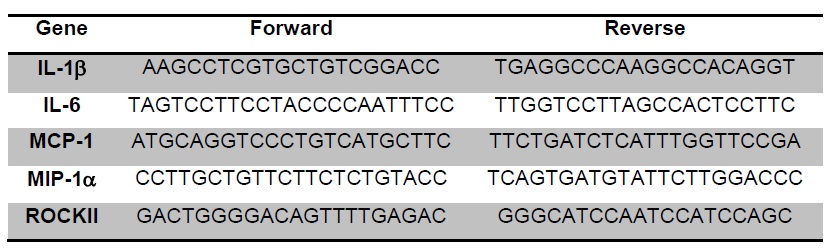


# Supplementary video

**Supplementary Video 1. LPS-induced microglial morphological dynamics affected by α-asarone treatment**

Video represents images captured from one field in a well of a 96-well plate. Images were captured at 10-minute intervals for 24 hours. Left: LPS-induced morphological dynamics; Right: α-asarone effect on the LPS-stimulated morphological dynamics.
